# Systemic challenges in AI adoption in public social and health organizations in Finland: a technology-organisation-environment perspective

**DOI:** 10.1108/JHOM-06-2025-0309

**Published:** 2025-09-30

**Authors:** Jarmo Pulkkinen, Kimmo Huttu, Marjo Suhonen

**Affiliations:** University of Lapland, Rovaniemi, Finland; Independent Researcher, Rovaniemi, Finland; Faculty of Social Sciences, University of Lapland, Rovaniemi, Finland

**Keywords:** Artificial intelligence, AI, Social and health organizations, Systemic challenges, TOE

## Abstract

**Purpose:**

The aim is to identify the key technological, organizational, and environmental challenges affecting the adoption of artificial intelligence (AI) in public social and health organizations. The Technology-Organization-Environment (TOE) theory was used as a framework for the study. As AI is increasingly utilized, research is needed to support organizational management and development work.

**Design/methodology/approach:**

We employed a mixed-methods research design, utilizing a web-based survey that included both quantitative, structured questions and qualitative, open-ended questions. The data included answers from experts within the Finnish social and healthcare AI innovation ecosystem (*n* = 82), representing public, private, and third-sector organizations. A theory-driven content analysis was conducted for the qualitative data, and descriptive statistical analysis was performed for the quantitative data.

**Findings:**

The challenges of AI adoption form a systemic whole where factors are strongly interdependent. All 46 challenges were rated at least somewhat significant (mean ≥ 1.6, scale 0–3), with an overall mean score of 2.05. Organizational challenges emerged as the most critical, notably limited financial resources, insufficient AI competence, and inadequate change management. Among the environmental challenges, ambiguity in legislative interpretation and national funding shortfalls were particularly notable. Experts with prior involvement in AI projects rated challenges statistically less substantial than those with less experience.

**Originality/value:**

This study provides the first national-level analysis examining AI adoption challenges across all three TOE theory dimensions in public social and healthcare organizations, empirically demonstrating their systemic interdependencies through multi-stakeholder perspectives. Previous research has primarily focused on specific AI applications or individual organizational factors.

## Introduction

Western public social and healthcare organizations face fundamental structural challenges that require novel solutions to ensure the sustainability of service provision. An aging population increases the demand for services, and the working-age population is shrinking (Äijö *et al*., 2022; Valkama and Oulasvirta, 2021). The scarcity of public finances further compounds these challenges. These factors create a complex situation for which established operational models are no longer adequate. In Finland, the 21 wellbeing services counties, created through the recent national public social and health services reform, are seeking new operational models to address the growing demand for services with limited resources (Tynkkynen *et al*., 2022).

Significant expectations are placed on advanced technologies, such as artificial intelligence (AI) functionalities and software, to solve some of these challenges (Konttila *et al*., 2019; Makarius *et al*., 2020; Mergel *et al*., 2023). For example, predictive models based on machine learning, natural language processing to automate administrative processes, and diagnostic technologies that enhance clinical work can improve resource allocation and streamline processes (Arslantaş, 2024; Graham, 2025; Wen and Huang, 2022). At its best, using advanced technologies leads to better productivity, employee job satisfaction, and higher-quality services (Mergel *et al*., 2023).

Despite the potential of AI, its adoption in, for example, Finnish public social and healthcare organizations has remained sporadic and limited to individual pilot projects (Niemelä *et al*., 2025; Una Oy, 2025). This reflects international experiences, where expected productivity gains have not always materialized (Liu *et al*., 2024) and AI adoption has encountered numerous technological, organizational, and institutional barriers (Campion *et al*., 2022; Neumann *et al*., 2024; Vatamanu and Tofan, 2025). Understanding these challenges is essential for realizing AI’s potential to benefit citizens, clients, patients, and social and healthcare professionals. This requires accountable policy steering to ensure that adoption processes align with societal values, professional standards, and the principles of equity.

Previous public sector AI research has focused mainly on individual AI applications, single organizations, the specific characteristics of the public sector, citizen perspectives, and productivity impacts (e.g., Brauner *et al*., 2023; Dreyling *et al*., 2024; Gökalp *et al*., 2025; Li and Wang, 2024; Liu *et al*., 2024; Mergel *et al*., 2023). Although recent studies have begun to recognize the systemic nature of AI adoption (e.g., Alami *et al*., 2021; Roppelt *et al*., 2024), national-level empirical analysis of how technological, organizational, and environmental factors interact, particularly within the context of public social and healthcare organizations, remains limited (Kumar *et al*., 2025; Neumann *et al*., 2024).

Our research asks: What are the most significant technological, organizational, and environmental challenges in adopting AI in public social and healthcare organizations? Finland provides an appropriate research context as it combines an AI pioneering status (IMF, 2023) with a recently reformed healthcare system organized around 21 wellbeing services counties. This setting provides unique insights into the challenges of AI adoption within a tax-funded universal social and healthcare system undergoing structural transformation. We collected the data in November–December 2024 via an online survey from experts (*n* = 82) within the national social and healthcare AI innovation ecosystem established by the Ministry of Social Affairs and Health (2025). Respondents comprehensively represented experts from the social and healthcare sector, including 17 of 21 wellbeing services counties, HUS Helsinki University Hospital, the City of Helsinki, and the private and third sectors. We use the Technology-Organization-Environment (TOE) framework (Tornatzky and Fleischer, 1990) as our theoretical lens. The TOE framework enables the examination of factors influencing the adoption of technological innovations within an organizational context. It is well-suited for analyzing challenges both separately and as a systemic whole, where different factors influence one another.

Digitalization in the public sector should indeed be viewed as a systemic phenomenon where technological, organizational, and environmental factors are intertwined (Haug *et al*., 2023; Mergel *et al*., 2019). Adopting technologies, such as AI software and functionalities, is not merely a technical process but a social and institutional transformation where governance structures, power dynamics, and organizational culture critically influence the outcome. Managing digital change is concurrently managing service transformation (Vial, 2019). From a systems thinking perspective, AI adoption requires understanding the systemic nature of change and the interrelationships between different factors (Bunge, 2004). Continuous technological development demands multi-level flexibility from public administration and service provision, and the ability to manage unpredictable changes (Haug *et al*., 2023; Mergel *et al*., 2019).

This study contributes to the scientific discourse on digital transformation and technology adoption in public social and healthcare organizations by providing an empirically grounded understanding of the systemic challenges in AI use within these organizations. A key theoretical contribution is the application of the TOE framework to examine AI adoption challenges in public social and healthcare organizations, demonstrating its utility for understanding systemic interdependencies in this context. As a practical contribution, we provide evidence-based insights to inform organizational development and management practices when introducing AI in public service settings.

The article proceeds from a review of previous research related to our research question and the theoretical framework, a description of methods and ethical considerations, the presentation of findings, discussion, conclusions, and recommendations. Lastly, we assess the reliability of the study.

### Previous research on the challenges of AI use in social and healthcare

Research on the utilization of AI in social and healthcare has been extensive over the past decade. In healthcare, research has concentrated on diagnostic applications, particularly medical imaging and radiology, gastroenterology, ophthalmology, and digital pathology, with studies demonstrating consistent technical achievements across diverse healthcare systems (e.g. Campanella *et al*., 2019; Esteva *et al*., 2017; Haenssle *et al*., 2018; Kather *et al*., 2019). Previous research has mainly focused on clinical and technical aspects rather than healthcare service delivery, organizational implementation processes, or the operational development challenges faced by healthcare organizations (Alnasser *et al*., 2024; Neumann *et al*., 2024). In contrast to the healthcare sector’s focus on diagnostic applications, AI research in social services remains in early stages (Garkisch and Goldkind, 2024).

Comparative studies reveal that the utilization of AI in public sector social and healthcare services has proven more challenging than in private healthcare, primarily due to the complex operating environment and specific regulatory requirements (Hassan *et al*., 2024; Morrison, 2021). Despite this extensive AI research activity, national-level empirical analysis of adoption challenges remains limited and fragmented, with most studies focusing on individual applications or single organizational contexts rather than a systematic examination of implementation barriers (Ali *et al*., 2023; Hassan *et al*., 2024; Neumann *et al*., 2024; Roppelt *et al*., 2024).

The challenges identified by previous research can be structured into three main, interdependent categories according to the Technology-Organization-Environment (TOE) framework (Tornatzky and Fleischer, 1990).

### Technological challenges

Technological barriers focus on the characteristics of AI technologies, their integration with existing systems, and data. Information system interoperability issues have proven to be a significant obstacle to connecting AI solutions with existing technological infrastructure (Saprudin, 2024). Data quality, quantity, and availability are critical bottlenecks for developing AI algorithms and end results, as insufficient or fragmented data can lead to erroneous or biased results (Ali *et al*., 2023).

The lack of algorithmic transparency, commonly referred to as the “black box” problem, is particularly challenging in the social and healthcare sector, where decisions must be justifiable to professionals, patients, and clients (Ghassemi *et al*., 2021; Schiff *et al*., 2022; Topol, 2019). The demand for explainability is further emphasized when AI is applied to clinical decisions. Data security and privacy protection constitute another key challenge when processing sensitive patient and client data. The rapid development of AI technology can create dependencies on external vendors and complicate an organization’s maintenance of technological competence (Koskimies *et al*., 2022; Neumann *et al*., 2024).

### Organizational challenges

Organizational challenges encompass various factors related to an organization’s internal structures, processes, resources, and culture (Makarius *et al*., 2020; Mergel *et al*., 2023). Insufficient AI competence among staff and management has been identified as a key barrier in several studies (Arboh *et al*., 2025; Neumann *et al*., 2024). This competence gap pertains not only to technical skills but also to understanding AI’s potential and limitations, as well as its application in practical work.

In the social and healthcare context, change management becomes particularly complex as it is deeply intertwined with professional identities, patient safety culture, and ethical considerations (Singh *et al*., 2020; Sun and Medaglia, 2019). AI can challenge established clinical workflows and professional autonomy, requiring particularly sensitive and participatory change management. Social and healthcare professionals have a strong ethical responsibility, and patient safety is central, which is why AI’s “black box” nature can conflict with these principles (Hua *et al*., 2024).

The lack of various resources is a challenge in the public sector, regarding both financial and human resources. AI projects can require significant initial investments and ongoing maintenance, which is challenging within the predetermined budgetary frameworks of public administration (Petersson *et al*., 2022). Complex decision-making structures and bureaucracy in public administration slow the adoption of new technologies (Dreyling *et al*., 2024; Mergel *et al*., 2023). Employee involvement in development processes has been identified as a critical factor for successful implementation (Neumann *et al*., 2024).

### Environmental challenges

Environmental challenges relate to factors external to the organization, including legislation, regulations, and national policies. The EU’s General Data Protection Regulation (GDPR) and the EU AI Act set significant boundary conditions for the use of AI, especially concerning the processing of personal data (Schiff *et al*., 2022). The ambiguity in interpreting these regulations and their practical application can cause uncertainty and slow down development projects (Floridi and Cowls, 2022).

The lack of national coordination and clear strategic guidelines has been identified as a significant challenge in several countries (Papyshev and Yarime, 2023; Sun and Medaglia, 2019; Wirtz *et al*., 2019). Without a clear national will and support, it is challenging for public organizations to advance the use of AI systematically. Ethical issues, such as non-discrimination, fairness, and accountability, require continuous reflection and guidance (Elendu *et al*., 2023; Niari, 2024). Citizens’ trust in and acceptance of AI-based services are also essential (Alhosani and Alhashmi, 2024; Ryan, 2020).

Environmental challenges are not passive obstacles. They can actively undermine organizations’ ability and willingness to invest in AI development. Legislative ambiguity and a lack of national coordination create uncertainty that can paralyze development (Wilson, 2022). Unclear regulations increase risk and reduce investment willingness, directly impacting the organizational context. Furthermore, collaboration challenges between actors, such as the public and private sectors, slow down AI development (Koskimies *et al*., 2022; Neumann *et al*., 2024).

### Theoretical framework

The theoretical framework for this study is the Technology-Organization-Environment (TOE) framework (Tornatzky and Fleischer, 1990). The TOE framework is an established model that provides a lens for understanding the adoption of technological innovations in an organizational context (Baker, 2012). Central to the framework are three dimensions that influence an organization’s decision-making and the successful adoption of an innovation: the technological, organizational, and environmental contexts. The TOE framework was utilized in this study for data collection (questionnaire design), data analysis (theory-driven content analysis), and the interpretations based on the analysis.

The technological context refers to the internal and external technologies relevant to the organization’s operations (Baker, 2012; Oliveira *et al*., 2011). This includes the innovation’s characteristics, such as its relative advantage over current solutions, compatibility with the organization’s technologies and practices, and ease of use and understanding. In AI, essential factors include data availability and quality, algorithm reliability and transparency, as well as concerns regarding data security and privacy.

The organizational context comprises the organization’s characteristics and resources that can promote or hinder the adoption of innovation (Baker, 2012; Oliveira *et al*., 2011). These include organizational size and structure, management support for the innovation, staff competence and skills, available financial and technological resources, internal communication and decision-making processes, and organizational culture. Strong management support and sufficient AI competence can significantly facilitate AI adoption.

The environmental context covers external factors affecting the organization’s operations and decision-making (Baker, 2012; Oliveira *et al*., 2011). These include industry structure, legislation and regulation, technology market developments, stakeholder pressures, and national policies and support measures. Legislation and ethical norms are crucial in the public social and healthcare sector.

The strength of the TOE framework lies in its ability to offer a comprehensive perspective on adopting technological innovations (Oliveira *et al*., 2011). It recognizes that no single dimension alone explains successful adoption. The dynamic interplay of these three levels is crucial. The framework has been successfully applied in research on various technologies and organizational types, including digital transformation in the public sector (Crossan and Apaydin, 2010; Oliveira *et al*., 2011).

## Materials and methods

The context of the study is public social and healthcare organizations in Finland, where taxes fund social and healthcare services, and public services are available to all citizens. An extensive reform was recently implemented to reform these services, transferring the organization of services from municipalities to 21 wellbeing services counties, the City of Helsinki, and the HUS Helsinki University Hospital (Tynkkynen *et al*., 2022). In recent years, AI has been implemented in social and healthcare services to ensure service availability and the efficient use of resources (Ministry of Social Affairs and Health, 2025; Niemelä *et al*., 2025).

We employed a mixed-methods research design, utilizing a web-based survey that included quantitative structured questions (46 challenges to AI adoption, assessed using a Likert scale) and qualitative, open-ended questions. This mixed-methods approach was selected because: (1) systematic assessment of multiple dimensions required standardized measurement across diverse organizational contexts; (2) examining TOE-framework's technological, organizational, and environmental factors simultaneously required a structured approach that surveys facilitate; (3) capturing perspectives from geographically dispersed stakeholders across Finland’s 21 wellbeing services counties made survey more practical than extensive interviews; and (4) both quantitative priority identification and qualitative contextual understanding were essential for the study.

### Data collection

Given that AI utilization in Finland’s social and healthcare sector is still in its early stages, we employed purposive expert sampling (Palinkas *et al*., 2015) to ensure respondents possessed relevant knowledge and experience to assess adoption challenges meaningfully. We identified participants from the Ministry of Social Affairs and Health’s (2025) national AI innovation ecosystem, as they represent the most knowledgeable stakeholders in this emerging field in Finland. This AI ecosystem was established in 2024 to coordinate AI development efforts and includes individuals with direct involvement in AI initiatives, policy development, or implementation support across the social and healthcare sector. This ecosystem encompasses representatives from all 21 wellbeing services counties, HUS Helsinki University Hospital, the City of Helsinki, relevant ministries, state agencies and institutions, third-sector organizations, research institutions, and private companies with demonstrated expertise in AI development for social and healthcare applications.

The expert sampling approach was essential because organizational-level AI adoption challenges require assessment by individuals with comprehensive exposure to the technological, organizational, and environmental dimensions of AI implementation. For example, frontline healthcare professionals possess critical perspectives on organizational challenges. Our research, however, focuses on systemic barriers across all dimensions of the TOE framework, requiring participants also with diverse expertise in technology development, policy implementation, organizational management, and regulatory environments. This organizational focus differentiates our research from clinical studies that examine individual AI tool adoption by healthcare professionals, as we investigated systemic adoption barriers and enablers at the organizational and institutional levels rather than the clinical utility of specific applications. The Ministry’s ecosystem registry provided the most comprehensive and relevant expert population available in Finland for this research purpose.

Data were collected (Nov–Dec 2024) through a web-based survey comprising 46 quantitative questions (Appendix) that addressed challenges in AI adoption. The questions were developed based on previous scientific literature and expert consultation through a focus group of eight specialists from wellbeing services counties, the Ministry, research institutions, and companies, conducted in October 2024. The questions were categorized according to the TOE framework into technological (15 questions), organizational (17 questions), and environmental (14 questions) categories. Background information about respondents was also collected, including their organization type, experience in adopting AI solutions in the public social and healthcare sector, AI technologies used, and from those working in wellbeing services counties, HUS Helsinki University Hospital, or the City of Helsinki, more detailed information about their service area and possible management roles.

In the questionnaire, the format for structured questions was: “*How would you assess the following [technological/organizational/environmental] challenge in the AI development of social or healthcare services in a wellbeing services county, HUS, or Helsinki?*”. Response options were on a four-point Likert scale: “Not a challenge at all” (value 0), “Minor challenge” (value 1), “Significant challenge” (value 2), and “Very significant challenge” (value 3). There was also an option “I cannot say,” which was not included in the mean calculations. Respondents could provide open-ended answers about the challenges and solutions for each TOE theme in connection with each TOE section.

## Respondents

Survey invitations were emailed to all registered experts (*n* = 930) in the Ministry of Social Affairs and Health’s AI innovation ecosystem. Eighty-two experts responded to the survey (response rate 8.8%). The largest respondent group was from wellbeing services counties (*n* = 32, 39%), followed by private software vendors and consulting firms (*n* = 13, 16%), and universities and public research institutions (*n* = 9, 11%). Eight individuals (10%) responded from the HUS Helsinki University Hospital, and five from private social and healthcare service providers. Other respondent entities included organizations and associations (*n* = 4), ministries and state agencies (*n* = 2), and other actors. The respondent group broadly represents the various actors in the AI ecosystem. However, the proportion of public organizations (17 wellbeing services counties, HUS Helsinki University Hospital, Helsinki, ministries, and agencies) is the largest. Approximately half of the respondents had been involved in implementing AI solutions, allowing for a comparison of experience-based views with those who lacked such experience.

### Data analysis

The quantitative data were primarily analyzed using descriptive statistical methods (Palinkas *et al*., 2015), such as means of Likert scale responses (Sullivan and Artino, 2013). The qualitative data (4,150 Finnish words from open-ended question responses) were analyzed using theory-driven content analysis (Elo and Kyngäs, 2008). The units of analysis were the conceptual entities related to challenges and solutions expressed by the respondents, which were classified under the three main categories of the TOE framework. The qualitative analysis aimed to deepen the quantitative results, highlight respondents’ experiences, and identify interconnections between challenges and their systemic nature.

### Ethics

Good scientific practice and ethical principles have been followed in conducting this research (ALLEA, 2023). An ethics committee statement was not sought for the study, as anonymous expert surveys on non-sensitive topics do not typically require it in Finland. The survey invitation was sent to members of the Ministry of Social Affairs and Health’s (2025) AI ecosystem register, adhering to appropriate permission procedures. One of the article’s authors worked within the ecosystem during the research period, but conducting this study was not related to their work tasks.

Participation in the study was voluntary. The cover letter for the survey explained the purpose of the research, the use of data in a scientific publication, and the voluntary nature of participation. In reporting the results, care was taken to ensure that respondents’ identities could not be identified from the published results. Current data protection legislation was followed in storing, processing, and archiving the data (European Parliament and Council, 2016).

## Findings

### The systemic nature of challenges

The challenges of AI adoption in Finnish public social and healthcare organizations form a complex, interdependent network. This systemic nature is evident, for example, in that all 46 identified challenges were assessed as at least moderately significant (mean ≥ 1.6), indicating that no single factor can be isolated from others (Appendix). The overall mean for challenges, 2.05 on a scale of 0–3, falls into the lower end of the “significant challenge” category. However, more important are the slight differences between categories: organizational factors have a mean of 2.16, environmental factors have a mean of 2.02, and technological factors have a mean of 1.94. Thus, the differences between categories were relatively small, supporting the TOE framework’s (Tornatzky and Fleischer, 1990) view of the interdependence of different dimensions in adopting technological innovations.

Systemic connections between categories were evident in the qualitative data. Legislative uncertainties in the operating environment directly impact organizations’ investment capacity and willingness, limiting the development of technological competence. Correspondingly, organizations’ lack of technological understanding reinforced fears related to environmental regulation and slowed down decision-making. This cycle was evident in assessments related to data security and privacy, where technical solutions existed, but organizational competence to apply them was lacking: “*For example, data protection and security are a very significant challenge for progress, but it is more of an organizational challenge than genuinely weak security of solutions*” (R81).

The interdependence of challenges is also highlighted by the fact that the three most significant individual obstacles (financial resources, technical AI competence, and availability of AI experts) can be considered to represent all TOE dimensions. These underlying factors feed each other: a shortage of experts raises costs, high costs limit recruitment, and a lack of competence weakens investment productivity.

Several technological factors, such as the availability of digital materials and the suitability of AI solutions, were among the least significant challenges.

### Differences between those who utilized AI and others

**Figure 1:**
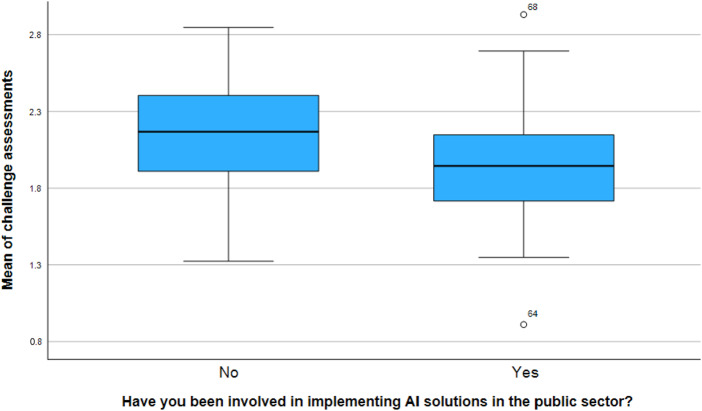
Those involved in adopting AI solutions assessed challenges as less significant on average than those who had not been engaged in utilization. Source: Authors’ own work

Experts who had participated in implementing AI solutions (*n* = 36) assessed the challenge statements as less significant on average than their inexperienced colleagues (*n* = 42), Figure 1. The difference in the mean of challenges between the groups was tested using an independent samples *t*-test, where the mean of challenge assessments for each respondent was calculated. The result showed a statistically significant difference (t(76) = −2.29, *p* = 0.025). The difference between the groups suggests that preconceived notions about challenges may be greater than obstacles. Based on open-ended question responses, experiments, and piloting can effectively reduce perceived challenge levels.

The impact of prior experience with AI use was evident in technological challenges, where experienced actors identified many obstacles as manageable problems rather than insurmountable limitations. Data security and privacy issues, which the inexperienced considered significant obstacles, appeared to the experienced more as procedural challenges than fundamental barriers.

Organizational challenges are closely linked to technological challenges … In my opinion, solutions have been found for all challenges (R81)

Experience had a particularly significant influence on the assessment of resistance to change in organizational challenges. Experienced actors identified resistance to change as a lesser problem, suggesting that involving personnel in the development process reduces opposition. Similarly, difficulties in the procurement competence and change management appeared more manageable to the experienced.

Prior AI experience had the least impact on the assessment of environmental challenges. This may be explained by the fact that legislative and national coordination issues affect all actors equally, regardless of their experience.

Software and system vendors assessed technological challenges as smaller than those of representatives from wellbeing services counties. This likely reflects their confidence in the technical functionality of their solutions, but may also indicate that vendors underestimate the specific requirements of public social and healthcare organizations.

However, this experience effect we found must be interpreted cautiously, given potential self-selection bias. Experienced respondents may represent early adopters who are inherently more optimistic about technology, work in better-resourced organizations, or have encountered relatively straightforward AI implementations.

### Multidimensional challenges related to technology

Although the mean for technological challenges (1.94) was the lowest when examined by TOE categories, this does not tell the whole truth about their significance for AI development. While the availability of digital materials (mean 1.6) and the suitability of AI solutions (mean 1.7) were perceived as the least significant obstacles, technical AI competence within organizations emerged as the second most crucial challenge in the entire study (mean 2.4). This paradox suggests that the problem is not a lack of technology, but rather employees’ ability to understand and apply it. Key technological challenges arising from the qualitative data are outlined in Table 1.

**Table 1:** Key technological challenges from the qualitative data

Specific technological barrier	Key impact	Empirical evidence
Lack of technical AI understanding among professionals	Cannot evaluate suitability or make informed decisions	“Few understand the operational logic of AI applications adequately” (R26)
Unclear security standards and guidelines	Prevents experimentation due to compliance uncertainty	“It has not been jointly agreed whether AI use is secure and data-protected” (R6)
Multiple incompatible information systems	AI solutions cannot connect to existing infrastructure	“several different information systems that do not communicate with each other” (R35)
Budget constraints favor the cheapest solutions	Limits organizational adoption capacity	“only the cheapest solutions are adopted- > resulting in minimal practical benefit” (R16)
Poor and fragmented data availability	Makes AI algorithm training difficult or impossible	“data quality and deficiency” (R56)
Speech recognition and transcription inadequacy	Reduces accuracy and applicability in the Finnish context	“Finnish language speech understanding and transcription” (R58)
Lack of safe testing infrastructure	Prevents proper AI validation before deployment	“lack of testing environments hinders technology adoption” (R22)
Vendor promises vs. real-world implementation	Organizations cannot operationalize promising solutions	“Vendors’ promotional speeches about technology readiness rarely meet the real world” (R37)

**Source(s):** Authors’ own work

Data security and privacy were assessed as one of the most significant technological obstacles to AI utilization (mean 2.2). Still, the analysis revealed these security issues to be more a matter of interpretation and competence than a technical constraint. Algorithmic transparency, the “black box” problem (mean 2.1), as well as ensuring patient safety (mean 2.1), reflect the specific requirements of the social and healthcare sector, where decisions must be justifiable to both professionals and patients/clients:

We do not have enough up-to-date and context-specific (Finnish social and healthcare field) research data on the technical aspects of AI to make ethically and economically sustainable decisions (R28).

Technical costs (mean 2.1) and challenges in transitioning to production after the pilot phase (mean 2.0) indicate that the success of AI projects depends as much on systematic implementation as on technical functionality. This reflects a broader problem in the gap between technological capability and organizational readiness in social and healthcare organizations.

### Organizational underlying factors

Organizational factors constitute the key underlying drivers in AI adoption. The availability of AI experts within organizations (mean 2.4), procurement competence (mean 2.4), and the allocation of employee resources to AI adoption emerged as the most significant organizational challenges. These challenges are closely intertwined with the need for change management (mean 2.3) and management’s understanding of AI’s strategic importance and the complexity of implementation processes (mean 2.3).

Top management is open-minded, but the support of line managers for developing and implementing new operating models is often insufficient. Resourcing employees for development work is also inadequate (R35).

Key organizational challenges arising from the qualitative data are outlined in Table 2.

**Table 2:** Key organizational challenges from the qualitative data

Specific organizational barrier	Key impact	Empirical evidence
Personnel time consumed by basic work	Prevents strategic development work and training	“Staff time goes largely to basic work, learning new skills always takes time” (R18)
Limited leadership comprehension of AI benefits	Inadequate support and resource commitment	“Top management does not understand how AI would concretely bring something new” (R18)
Price-focused rather than value-based purchasing	Results in inadequate technology that provides little value	“only the cheapest solutions are adopted” (R16)
Hierarchical and inflexible work culture	Creates years-long delays in implementation decisions	“Social and healthcare culture is still hierarchical and rigid” (R21)
Fear-based attitudes toward new technology	Staff avoid adoption due to concerns about competence	“Attitude against everything new unfortunately often stems from fear of not knowing how” (R2)
Insufficient qualified professionals available	Cannot recruit, develop, or retain necessary talent	“I don’t believe sufficient expertise can be found within wellbeing services counties” (R18)
Weak technology competence in social care	Prevents effective utilization of AI tools	“on the social care side, digital and technology competence is really minimal generally” (R28)
Poor collaboration between IT and clinical staff	Creates implementation bottlenecks and conflicts	“ICT administration is quite far from service production and communication seems limited” (R29)

**Source(s):** Authors’ own work

The allocation of personnel’s working time to AI adoption (mean 2.4) reflects a broader need to develop resource allocation in the social and healthcare sector. Changes caused by the social and healthcare service reform implemented in Finland may have reduced organizations’ ability to allocate resources to development work, as there are dwindling financial and personnel resources for coping with basic operations.

Wellbeing services counties are currently in such turmoil with the social and health care service reform that there is hardly any time for development. This means that not only internal organizational dialogue, learning, and co-development but also inter-organizational efforts are on hold due to lack of time and resources (R52).

Sector-specific differences in social and healthcare were particularly prominent in the area of competence, where technology competence in social care was assessed as weaker than in healthcare. This reflects the different technological histories and educational traditions of the sectors.

Generally, professionals in the Finnish social and healthcare field are interested in AI, but the problem is that many do not have sufficient knowledge and competence to assess the suitability of AI because few understand the operational logic of AI applications adequately. Especially in social care, digital and technological competence is generally very low, with a few exceptions (R28).

Employee resistance to change, however, was assessed as a lesser organizational challenge (mean 1.8). This suggests that the problem lies not so much in attitudes but in organizational readiness and resources. Aligning professional groups’ work practices with AI (mean 2.2) and developing digital skills (mean 2.0) requires systematic competence development.

Competence in applying legislation within organizations (mean 2.3) is challenging, as it requires combining technological understanding with the interpretation of legal matters. This need for dual competence makes AI development particularly demanding in public social and healthcare organizations.

### Institutional barriers in the operating environment

The operating environment’s challenges were divided into three main themes: legislative uncertainties, national coordination shortcomings, and inter-actor collaboration problems. These themes formed an interconnected system where coordination failures amplified legislative uncertainties and constrained collaboration opportunities. The systemic nature of environmental challenges became evident in their mutual reinforcement: legislative uncertainty reduced investment willingness, limited coordination prevented knowledge sharing, and resource constraints amplified competitive pressures for expertise. Restrictions related to the processing of personal data (mean 2.1) and the ambiguity of national legislation (mean 2.1) were key challenges arising from the operating environment.

Key environmental challenges arising from the qualitative data are outlined in Table 3.

**Table 3:** Key environmental challenges from the qualitative data

Specific environmental barrier	Key impact	Empirical evidence
Ongoing organizational changes are consuming resources	Prevents focus on development and innovation initiatives	“Wellbeing services counties are in such turmoil with the national reform that there’s hardly time for development” (R52)
Unclear guidance on regulation compliance	Creates risk-averse behavior and implementation delays	“there are problems in interpreting legislation and uncertainty in connecting new products” (R25)
Complex and ineffective data access system	Blocks research and algorithm development projects	“Findata currently does not serve at all what it was established for” (R29)
Insufficient public national investment in AI development	Limits organizational capacity for innovation	“Money is always the big problem” (R17)
Massive divide between healthcare and vendors	Results in unsuitable commercial solutions	“massive gap between public social and healthcare actors and private application vendors” (R64)
Limited vendor knowledge of public sector constraints	Products fail to address real organizational requirements	“System vendors don’t always have an understanding of public sector operations and needs” (R1)
Regulations preventing the use of patient data for AI training	Blocks the development of contextualized AI models	“Regulatory situation prevents AI training with real customer and patient data” (R61)
Price-focused and rigid acquisition procedures	Favors traditional vendors over innovative solutions	“procurement processes are so rigid and favor price competition and traditionally used operators” (R2)

**Source(s):** Authors’ own work

The impacts of the EU’s AI Act were assessed as significant (mean 2.0), but the analysis revealed that the problem lay more in the lack of application guidance than in the regulation itself. Challenges related to using the Findata (national health data permit authority) service (mean 2.2) reflect a broader problem with the complexity and slowness of national data services. This technical challenge interacted with legislative uncertainties to create compounded barriers for organizations seeking to comply with data processing requirements.

I do not believe that legislation will ultimately become a significant challenge, provided that clearer national interpretation and operational guidelines, i.e., guidance, are made available (R81).

The lack of national coordination and guidelines (mean 1.9) and insufficient national AI funding (mean 2.3) create an operating environment where organizations must function without clear strategic national support. This coordination deficit operated as a root amplifier, forcing individual organizations to navigate regulatory complexity and compete for limited resources independently. This is particularly emphasized in collaboration between wellbeing services counties (mean 1.9) and co-development with the private sector (mean 1.9).

The availability of AI experts in the workforce (mean 2.3) reflects a national challenge, as the public sector competes with the private sector for these highly skilled professionals. System vendors’ understanding of public sector needs (mean 2.0) suggests a need for improved collaboration across different stages of procurement and development. These factors created a reinforcement cycle in which limited expertise constrained organizations’ ability to effectively engage with vendors, while vendors’ limited understanding of public sector requirements reduced the suitability of their solutions.

There are major challenges in public-private sector collaboration, but not necessarily between social and healthcare actors. Rather, there is a massive gap between public social and healthcare actors and private application vendors. Social and healthcare actors do not have practical opportunities to procure every possible device (R64).

## Discussion

This study examined the technological, organizational, and environmental challenges associated with the adoption of AI in Finnish public social and healthcare organizations. It aimed to construct an empirically grounded understanding of the nature and interconnections of these challenges and evaluate the suitability of the Technology-Organization-Environment (TOE) framework (Tornatzky and Fleischer, 1990) for researching this complex phenomenon.

Our findings contribute to the AI adoption literature by empirically validating and extending the applicability of the TOE framework in public social and healthcare contexts. The main finding is that AI adoption challenges form a systemic whole in which the different TOE dimensions are tightly interconnected, with relatively small mean differences between technological, organizational, and environmental challenges. This confirms Tornatzky and Fleischer’s (1990) proposition that the three dimensions operate as systemic amplifiers. However, we extend existing knowledge by demonstrating that organizational challenges emerge as more prominent than technological ones when technologies are available, contradicting digital transformation literature that emphasizes technological barriers as primary obstacles. This supports previous studies highlighting the systemic nature of digitalization in the public sector (Alnasser *et al*., 2024; Armenia *et al*., 2021; Haug *et al*., 2023; Vial, 2019).

While experienced AI practitioners rated technological and organizational challenges as less significant, environmental challenges remained equally problematic regardless of experience level. This reveals that environmental factors represent structural barriers requiring coordinated policy intervention, whereas technological and organizational challenges can be addressed through individual organizational learning. This finding suggests that preconceptions, lack of experiences, and fears may be greater obstacles than technical challenges, supporting the importance of experimentation, pilots, and knowledge sharing in dispelling uncertainties and accelerating learning.

Among environmental challenges, the ambiguity of legislation and inadequate national coordination and funding emerged as particularly significant. This reinforces findings from previous studies that public sector AI development requires strong national guidance and a clear legislative framework (Hassan *et al*., 2024). Specifically, our results indicate how insufficient national funding, environmental regulation ambiguities, and inadequate coordination impact organizations’ ability to address technological challenges and develop necessary competence. This demonstrates that AI adoption challenges are highly context-dependent, varying considerably across healthcare organizations based on their specific resources, capabilities, and operating environments.

Although AI technologies are developing rapidly, their broader adoption appears to be hindered more by organizational and institutional challenges than by purely technological constraints. The lack of technical AI competence emerged as the second most significant challenge overall, despite technological challenges being rated least critical. At the same time, the availability of digital materials was perceived as one of the least significant challenges. This suggests that the most acute technological challenges lie not in the technology itself or data availability, but in human and organizational capabilities required to utilize it. These organizational barriers align with Storey and Buchanan’s (2008) analysis of healthcare management and organizational barriers to new learning.

The theoretical contribution of this study provides empirical evidence for the applicability of the TOE framework in analyzing AI transformation challenges in complex public social and healthcare fields. We demonstrated that successful AI adoption requires simultaneous intervention across all TOE dimensions rather than sequential approaches, as environmental constraints can paralyze organizational investment capacity while weak organizational readiness limits effective technology utilization. Drawing on Bunge’s (2004) systemic perspective, technologies should be understood not as isolated entities, but as components within dynamic systems of social, structural, and functional interdependence. This means shifting from generalized AI discourse to process-based analyses of applied technological systems and their concrete effects in organizational contexts (Hanelt *et al*., 2021; Marques and Ferreira, 2020; Tortorella *et al*., 2019). This highlights the crucial need for public administration research to make these systemic challenges visible and provide evidence-based insights for policy development and cost-effective organizational management.

While this study focuses on Finland, the findings are likely to apply to countries with comparable healthcare characteristics, particularly those with tax-funded universal healthcare systems, such as the Nordic countries, Canada, and the UK. However, Finland's specific organizational structures and recent healthcare reform may limit direct transferability to nations with different financing models or regulatory environments. Future comparative research across diverse social and healthcare systems would enhance understanding of how national contexts shape AI adoption challenges.

## Conclusions

Our research indicates that addressing the challenges of AI utilization in Finnish social and healthcare services requires multi-level and coordinated measures. National-level coordination and an enabling operating environment must support organizations’ development work, enabling individual-level competence development and the adoption of new, AI-utilizing practices. This systemic perspective complements previous research, which has often focused on individual aspects of AI implementation (Busuioc, 2021; Grimmelikhuijsen, 2023; Ingrams *et al*., 2022).

National-level actions form the backbone of AI development, enabling progress at the organizational level. Legislators and ministries must actively create a more straightforward national strategy and roadmap for AI development in the public social and healthcare sector. From managing digital transformation, a key challenge is overcoming fragmentation and localization through systemic interaction in a spirit of co-development. Based on our research data, the importance of common national goals is particularly emphasized in the coordinated allocation of financial resources, a national recruitment strategy for AI experts, the optimization of human resources, and the sharing of best practices in change management. This strategic groundwork supports the findings of Mergel *et al*. (2023) on the specific characteristics of public sector AI development.

Addressing legislative uncertainties and improving national coordination requires institutionalized action through Finland’s existing AI governance structures. The Ministry of Social Affairs and Health should leverage, for example, the national AI innovation ecosystem to establish dedicated task forces that develop sector-specific EU AI Act implementation guidelines and facilitate systematic knowledge sharing between wellbeing services counties. This coordinated approach should include standardized AI readiness assessment tools, shared procurement frameworks, and centralized consultation services to prevent fragmented development efforts. This legislative clarification aligns with the recommendations of Schiff *et al*. (2022) and Tang *et al*. (2023). Critical infrastructure improvements also require national attention. The Findata service (national health data permit authority) requires streamlined processes and expedited approval pathways for AI projects, while sandbox environments within the national ecosystem would facilitate the safe testing of AI solutions with real-world data.

Long-term and sufficient funding for AI development, research, and adoption in the public social and healthcare sector must be secured, including investments in national competence development programs. At the national level, collaboration between wellbeing services counties, HUS Helsinki University Hospital, the city of Helsinki, companies, research institutions, and third-sector actors must also be actively supported and facilitated to avoid overlapping work and disseminate best practices.

At the organizational level, systematic competence development is crucial to the success of AI development. Organizations must prioritize the development of personnel’s AI competence and literacy at all levels, from top management to frontline employees. This supports the findings of Konttila *et al*. (2019) and Neumann *et al*. (2024) regarding the key role of competence. A broader understanding of AI’s possibilities and limitations in one’s work is needed. Management must commit to change, ensure sufficient resources for development work, and actively involve personnel in the design and implementation of AI solutions. This strengthening of change management aligns with the socio-technical perspective of Makarius *et al*. (2020). Organizations must formulate clear AI strategies and concrete goals integrated into their operations and development. Promoting a culture of experimentation and learning within organizations encourages trials and pilots, from which lessons are learned and experiences shared. This can help dispel uncertainties and identify the most suitable solutions.

The role of every public social and healthcare professional is central in identifying and developing needs-based technology solutions. The starting point should be a profound understanding of client and patient needs, as well as the desired social and structural impacts on service provision. Professionals can best assess where AI would bring added value to work processes and which problems primarily require solutions. Technology experiments and implementations should be based on the needs of overall architectural development, rather than technology dictating work methods (Niemi and Pekkola, 2020).

Future research should employ alternative methodological approaches to address the limitations identified in this study and enhance reliability through triangulation. Public administration research is particularly crucial for advancing our understanding of these complex organizational transformations, as systemic AI adoption challenges require investigation from governance, policy implementation, and organizational management perspectives. Ethical implications and patient trust mechanisms require systematic investigation, particularly examining how patient perceptions of AI transparency and fairness influence service utilization and healthcare professionals’ decision-making processes. Deeper methodological exploration is needed through in-depth follow-up interviews with survey respondents to provide a richer contextual understanding of the systemic interdependencies we have identified. Meanwhile, mixed-method case studies comparing successful and unsuccessful AI implementations could reveal how TOE factors interact in practice. Longitudinal studies tracking the same organizations over time would separate experience effects from organizational capacity differences, addressing concerns about self-selection bias in cross-sectional expert surveys.

The quantitative framework developed in this study provides a foundation for comparative analyses across national social and healthcare contexts, offering insights into how cultural, regulatory, and organizational factors influence the adoption of AI. Cross-national studies examining different healthcare funding models and regulatory approaches would enhance understanding of how these structural factors shape AI implementation outcomes across diverse social and healthcare systems.​​​​​​​​​​​​​​​

### Study limitations and quality assurance

The reliability and validity of this study have been ensured through several methodological choices. The questionnaire’s content validity was enhanced through expert consultation with a focus group of eight specialists from wellbeing services counties, the Ministry, research institutions, and companies. This pre-testing process enhanced the clarity, relevance, and comprehensiveness of the questions before the main data collection. The study’s credibility is further supported by the suitability of the data collection method (expert survey) for addressing the research question, which aimed to map the challenges of AI adoption comprehensively. Combining quantitative and qualitative data enhanced the depth and comprehensiveness of the results. The expertise of the respondents, as members of the social and healthcare AI ecosystem, and the rich and high-quality open-ended responses, support the credibility of the interpretations made. The study’s consistency and conditions for assessing transferability have been supported by describing the analysis process (theory-driven content analysis for qualitative data and descriptive statistical methods for quantitative data) and the entire research process transparently. The respondent group’s description helps the reader contextualize the results and assess their potential transferability to similar environments.

However, the study also has limitations. The survey response rate (approximately 8.8%) and the number of respondents (*n* = 82) are relatively low, which may limit the statistical generalizability of the results to the entire expert group within the social and healthcare AI ecosystem and general social and healthcare sector experts. This low response rate may partly reflect the early-stage nature of AI adoption in Finnish public social and healthcare, where many ecosystem members lack sufficient practical experience to feel qualified to respond meaningfully. However, this response rate is consistent with expert surveys in specialized fields (Baruch and Holtom, 2008). The quality and expertise of respondents may be more critical than quantity for our research objectives. The responding experts demonstrated high engagement, providing detailed qualitative responses (4,150 words total) that suggest thoughtful consideration of the questions. In addition, respondents broadly represented the ecosystem’s diversity, with 17 of 21 wellbeing counties participating, along with Helsinki University Hospital, the city of Helsinki, multiple ministries, research institutions, and private sector organizations. This comprehensive stakeholder representation supports the validity of the findings, despite the low response rate.

Our findings must be interpreted in consideration of the potential self-selection bias inherent in expert surveys. The voluntary nature of participation likely attracted individuals with more substantial interest in AI development, potentially leading to more optimistic assessments than would be observed in the general population of healthcare professionals. The finding that experienced AI practitioners rated challenges as less significant could reflect survivor bias: those with successful AI experiences may have worked in better-resourced organizations or encountered fewer barriers initially. However, the systematic differences between public and private sector respondents validate expected variations in challenge perception. At the same time, the stability of the results when excluding extreme responses suggests that outliers do not significantly influence our findings. The self-selection of AI-interested respondents may strengthen, rather than weaken, our conclusions about systemic challenges. If even AI-enthusiastic experts rate technical, organizational, and environmental challenges as significant (means > 1.9), this suggests that barriers are substantial enough to concern those most motivated to overcome them. This interpretation aligns with qualitative responses, indicating that even experienced practitioners encounter systemic obstacles that extend beyond individual projects.

Additionally, some respondents reported answering the survey as a group effort on behalf of their organization (consensus answer). While this may increase the comprehensiveness of an individual response from the organization’s perspective, it may also smooth out individual extreme views and mask internal tensions or disagreements within the organization. The Finnish context may limit the generalizability of findings to healthcare systems with different organizational or financing models. Due to these factors, caution must be exercised in interpreting the results, and they primarily represent the views of the expert group that responded to the survey.

Another limitation relates to the timing of the study. The data were collected at the end of 2024, only two years after the national public social and healthcare reform. This may have emphasized organizational and environmental challenges, such as resource shortages and general pressure for organizational change. Furthermore, the study is based on respondents’ self-assessments, which may contain subjective interpretations and emphases.

Despite these limitations, the study yielded valuable and timely insights from a diverse group of experts on the challenges of AI adoption in Finnish public social and healthcare organizations. The strategies employed to enhance reliability, including careful questionnaire design, the combination of quantitative and qualitative data, and a transparent description of the analysis process, strengthen the study’s scientific value.

## Appendix

**Table A1:** Mean values of all structured response options (T: Technological, O: Organizational, E: Environmental)

Order	Challenge	Mean of responses
#1	O: Financial resources	2.6
#2	T: Technical AI competence in social and healthcare organizations	2.4
#3	O: Availability of AI experts in social and healthcare organizations	2.4
#4	O: Procurement expertise for AI solutions	2.4
#5	O: Staff time availability for AI adoption and training	2.4
#6	O: Change management in processes and projects	2.3
#7	O: Understanding and support from management and decision-makers	2.3
#8	O: Competence in applying legislation within social and healthcare organizations	2.3
#9	E: Availability of AI experts in the workforce	2.3
#10	E: National AI funding	2.3
#11	O: Management commitment and securing resources for AI adoption	2.2
#12	E: Uniform practices for AI utilization in public social and healthcare services	2.2
#13	E: Challenges related to the use of the Findata service (national health data permit authority)	2.2
#14	T: Information security and data protection of AI solutions	2.2
#15	O: Challenges in assessing and measuring the impacts of safe AI use	2.2
#16	O: Reconciling current working methods of different professional groups with AI	2.2
#17	E: Restrictions related to the processing and use of personal data	2.1
#18	T: Transparency of AI algorithms (the so-called black box problem)	2.1
#19	T: Ensuring patient and client safety in AI solutions	2.1
#20	E: Limitations of national legislation in AI utilization	2.1
#21	T: Technical costs of AI solutions (implementation and maintenance)	2.1
#22	T: Challenges in transitioning AI solutions to production after the pilot phase	2.0
#23	O: Digital skills of healthcare and social welfare staff	2.0
#24	O: Building a technology-positive organizational culture	2.0
#25	E: System vendors’ understanding of public sector needs	2.0
#26	O: Identifying needs and utilization opportunities	2.0
#27	O: Developing IT management collaboration within social and healthcare organizations	2.0
#28	E: Impacts of the EU AI Act	2.0
#29	O: Systematic planning and phasing of AI adoption	2.0
#30	E: Co-development with private healthcare and social welfare actors	1.9
#31	T: Accuracy and reliability of AI solutions	1.9
#32	T: Ethics of AI solutions (e.g., ensuring non-discrimination and accountability)	1.9
#33	E: Cooperation between wellbeing services counties	1.9
#34	T: Technical customizability and compatibility of AI solutions with the current technological infrastructure of social and healthcare organizations	1.9
#35	T: Suitability of international AI solutions for Finnish social and healthcare services	1.9
#36	E: National coordination and guidelines	1.9
#37	E: Limitations of tendering and procurement legislation	1.9
#38	E: Citizens’ trust in AI-assisted social and healthcare services	1.8
#39	O: Developing an AI strategy	1.8
#40	O: Staff resistance to change regarding AI	1.8
#41	T: Trust in cloud-based AI solutions	1.7
#42	E: Citizens’ readiness to utilize AI-based solutions	1.7
#43	T: Suitability of AI solutions for the public social and healthcare sector	1.7
#44	T: Awareness of AI solutions and applications available on the market	1.7
#45	T: Suitability of AI solutions for different client and patient situations	1.7
#46	T: Availability or sufficiency of digital materials (e.g., patient or client data)	1.6

**Source(s):** Authors’ own work
